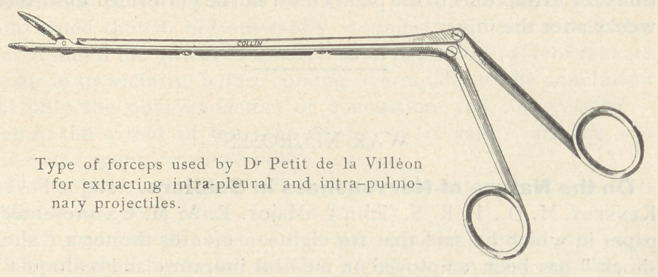# Removal of Foreign Bodies from the Chest

**Published:** 1918-08

**Authors:** E. Petit de la Villéon


**Extraction of Projectiles from the Pleura and Diaphragm.** By Doctor E. Petit de la Villéon.

The speaker presented conclusions based upon 60 operations for projectiles in the pleura and 16 for projectiles in the diaphragm. He hopes his experience may throw light upon the choice of technic in such cases, which, he considers, are among the most interesting problems of the war. He believes that the technic which he recommends affords a maximum of security and a minimum of useless surgical elaboration.

He early abandoned the classic method of thoracotomy in extracting projectiles in the pleura for the simpler method by means of forceps of his own design manipulated under a radioscopic screen and inserted through a small incision. The results thus obtained have been uniformly excellent with a minimum of surgical disturbance and no pneumothorax.

Extensive surgery of the walls and lungs he considers useless so far as healing is concerned; and he regards projectiles lodged in the pleura as essentially mobilizable and therefore disconcerting during an operation, for they change their position in a way to render thoracotomy difficult. When, however, the method of extraction by forceps is employed, the missile may be brought to its normal position by simple manipulation. The X-ray is an indispensable aid in all such work, and a topographic localization of the projectile must be made before any attempt to operate.

The speaker considers this technique equally satisfactory for all pleuro-parietal and pleuro-pulmonary positions of the projectile. Only when the mediastinal pleura are involved does the author consider a large thoracotomy necessary because of the extreme danger of piercing one of the large vessels of the hilum and in order to allow free access and lighting.

In extracting by forceps under a screen the author advises the invariable choice of an extremely oblique direction of approach, with the incision for entry at a considerable distance from the position of the projectile in the pleura. In this way the ribs will not inside the movements of the forceps. To remove a fragment lodged under the papilla, the incision (1 cm.) for entrance should be made in the anterior axillary region, cutting the *skin* only; and to reach a missile in the posterior pleura under the omoplate, the entrance incision should be made in the posterior axillary region. In case the rounding of the lung itself intervenes between the entrance incision and the projectile to be reached, the lung may be depressed by the forceps sufficiently to permit the projectile to be reached through the cavity. Formerly the speaker in

Type of forceps used by Dr Petit de la Villéon for extracting intra-pleural and intra-pulmonary projectiles.

such cases reached the foreign body by piercing the lung, but he has found such traumatism to be useless, even if it is not dangerous. He states that he is not concerned in this note with suppurating pleura, in which case wide resection and cleansing are necessary.

The speaker has also extracted over 250 intra-pulmonary projectiles by this method; here however, a more exact localization is necessary and care must be taken to assure oneself that the projectile is not in the region of the hilum.

*Projectiles in the diaphragm* may be reached by various methods accordingly as they are in the right, the left, or the median region. If the projectile is in the right portion of the diaphragm, it can be best reached by the forceps method through an incision made in the thoracic wall allowing the forceps to pass through the pleural cavity. In this region the diaphragm rests upon the liver " as upon a table ", facilitating the operation not a little.

When the missile is in the left part of the diaphragm, however, the conditions are very different, owing to the proximity of the stomach and of the splenic flexure of the colon. In such instances an open operation, preferably by the abdominal route is desirable. An oblique left laparotomy along the costal border is the method recommended. With the use of spreaders, the hand may be admitted freely, and the presence of the missile easily located without the aid of the X-ray.

Similarly in the medial region of the diaphragm, an open operation is requisite. In this case the use of the forceps through a small incision would be folly. For missiles in the phrenitic center or in the posterior region of the diaphragm, the author recommends a high laparotomy, sub-ombilical, medial or oblique.

The writer adds, however, that he does not consider the removal of all projectiles in this region as absolutely necessary. Operations for extractions in the pleura must not be performed until three weeks after the injury.

## Figures and Tables

**Figure f1:**